# Safety, tolerability and effects on cardiometabolic risk factors of empagliflozin monotherapy in drug-naïve patients with type 2 diabetes: a double-blind extension of a Phase III randomized controlled trial

**DOI:** 10.1186/s12933-015-0314-0

**Published:** 2015-12-23

**Authors:** Michael Roden, Ludwig Merker, Anita Vedel Christiansen, Flavien Roux, Afshin Salsali, Gabriel Kim, Peter Stella, Hans J. Woerle, Uli C. Broedl, On behalf of the EMPA-REG EXTEND™ MONO investigators

**Affiliations:** Department of Endocrinology and Diabetology, Medical Faculty, Heinrich Heine University Düsseldorf, Düsseldorf, Germany; Institute for Clinical Diabetology, German Diabetes Center, Leibniz Center for Diabetes Research, Düsseldorf, Germany; German Center for Diabetes Research, Partner Düsseldorf, Düsseldorf, Germany; Diabetes- und Nierenzentrum, Dormagen, Germany; Boehringer Ingelheim Danmark A/S, Copenhagen, Denmark; Boehringer Ingelheim France S.A.S., Reims, France; Boehringer Ingelheim Pharmaceuticals, Inc., Ridgefield, CT USA; Boehringer Ingelheim Pharma GmbH & Co. KG, Ingelheim, Germany

**Keywords:** Type 2 diabetes, Empagliflozin, SGLT2 inhibitor, Safety, Blood pressure

## Abstract

**Background:**

To investigate the long-term efficacy and safety of empagliflozin monotherapy compared with placebo and sitagliptin in drug-naïve patients with type 2 diabetes mellitus.

**Methods:**

Of 899 patients randomized to receive empagliflozin 10 mg, empagliflozin 25 mg, placebo, or sitagliptin 100 mg once daily for 24 weeks, 615 continued in a double-blind extension trial for ≥52 weeks. Exploratory endpoints included changes from baseline in HbA1c, weight and blood pressure at week 76.

**Results:**

Compared with placebo, adjusted mean changes from baseline in HbA1c at week 76 were −0.78 % (95 % CI −0.94, −0.63; p < 0.001) and −0.89 % (95 % CI −1.04, −0.73; p < 0.001) for empagliflozin 10 mg and 25 mg, respectively. Compared with placebo, adjusted mean changes from baseline in weight at week 76 were −1.8 kg (95 % CI −2.4, −1.3; p < 0.001) and −2.0 kg (95 % CI −2.6, −1.5; p < 0.001) for empagliflozin 10 mg and 25 mg, respectively. Empagliflozin led to reductions in systolic blood pressure (SBP) compared with placebo in the primary analysis but not in sensitivity analyses. Compared with sitagliptin, empagliflozin 25 mg reduced HbA1c and both empagliflozin doses reduced weight and SBP. Adverse events (AEs) were reported in 76.8, 78.0, 76.4 and 72.2 % of patients on empagliflozin 10 mg, empagliflozin 25 mg, placebo and sitagliptin, respectively. Confirmed hypoglycaemic AEs (glucose ≤3.9 mmol/l and/or requiring assistance) were reported in two patients (0.9 %) per treatment group.

**Conclusions:**

Empagliflozin monotherapy for ≥76 weeks was well tolerated and led to sustained reductions in HbA1c and weight compared with placebo.

Trial registration: clinicaltrials.gov NCT01289990

**Electronic supplementary material:**

The online version of this article (doi:10.1186/s12933-015-0314-0) contains supplementary material, which is available to authorized users.

## Background

Inhibition of the sodium glucose cotransporter 2 (SGLT2), located in the proximal tubule of the kidney, leads to increased urinary glucose excretion (UGE) and a reduction in plasma glucose levels in patients with type 2 diabetes mellitus (T2DM) [1–4]. This mechanism of action is associated with a low risk of hypoglycaemia, with additional benefits of weight loss and reductions in blood pressure [1, 5, 6].

Empagliflozin is a potent and selective SGLT2 inhibitor [7], which, when given as monotherapy or as add-on therapy for T2DM, has consistently reduced HbA1c, weight and systolic blood pressure (SBP) compared with placebo [8–16]. As well as reducing fasting plasma glucose (FPG), empagliflozin reduces post-prandial glucose in patients with T2DM [17].

In a Phase III, parallel-group, randomized, double-blind trial in drug-naïve patients with T2DM (EMPA-REG MONO™), empagliflozin 10 mg and 25 mg and the dipeptidyl peptidase-4 (DPP-4) inhibitor sitagliptin 100 mg given as monotherapy for 24 weeks were well tolerated and improved glycaemic control. Adjusted mean differences versus placebo in change from baseline in haemoglobin A1c (HbA1c) at week 24 were −0.74 % for empagliflozin 10 mg and −0.85 % for empagliflozin 25 mg, with no significant difference in change from baseline in HbA1c between empagliflozin and sitagliptin. Treatment with empagliflozin also significantly reduced body weight and SBP compared with placebo and sitagliptin [9].

This 52-week extension to the above study, EMPA-REG EXTEND™ MONO, evaluated the long-term safety, tolerability and efficacy of empagliflozin 10 mg and 25 mg compared with placebo and sitagliptin as monotherapy in patients with T2DM.

## Methods

### Study design

In the initial 24-week study, drug-naïve patients with T2DM (no oral or injectable anti-diabetes therapy for ≥12 weeks prior to randomization) with insufficient glycaemic control despite a diet and exercise regimen (HbA1c ≥7 to ≤10 %, or HbA1c ≥7 to ≤9 % in Germany) and body mass index ≤45 kg/m^2^ were enrolled. Key exclusion criteria included uncontrolled hyperglycaemia (glucose concentration >13.3 mmol/l following an overnight fast, confirmed by a second measurement), an estimated glomerular filtration rate (eGFR) [Modification of Diet in Renal Disease (MDRD) formula] <50 ml/min/1.73 m^2^, indication of liver disease, and contraindications to sitagliptin according to the local label [9].

In the initial study, patients were randomized (1:1:1:1) to receive empagliflozin 10 mg, empagliflozin 25 mg, sitagliptin 100 mg, or placebo once daily for 24 weeks. Patients who completed 24 weeks’ treatment, who still did not contravene the exclusion criteria for the initial study, and who did not contravene additional exclusion criteria for the extension study, e.g., eGFR <30 ml/min at the last visit of the initial trial, could decide to continue their double-blind treatment for ≥52 weeks (i.e., a total treatment duration of ≥76 weeks). Patients remained on the treatments they received in the initial study, but were required to re-confirm their consent before starting the extension trial. The initial trial and the extension trial were registered with clinicaltrials.gov (NCT01177813 and NCT01289990, respectively) and were carried out in compliance with the protocols and the principles of the Declaration of Helsinki, and in accordance with the International Conference on Harmonization Harmonized Tripartite Guideline for Good Clinical Practice. The studies were approved by Institutional Review Boards, Independent Ethics Committees and Competent Authorities according to national and international regulations.

During the extension trial, patients continued to receive diet and exercise counselling based on local recommendations. Patients who received rescue medication during the initial 24-week study and were still receiving it at the start of the extension study were to continue their rescue medication throughout the extension study. Rescue medication could be initiated during the extension trial if a patient had a confirmed plasma glucose level >10 mmol/l after an overnight fast or HbA1c >8 %. The choice and dose of rescue medication were at the discretion of the investigator, except that DPP-4 inhibitors and glucagon-like peptide-1 analogues were not permitted. In cases of hypoglycaemia, dose reduction or discontinuation of rescue medication was to be initiated. If hyper- or hypoglycaemia could not be controlled, the patient was to be discontinued from the trial.

### Endpoints and assessments

The primary efficacy endpoint of change from baseline in HbA1c at week 24 was analyzed in the initial trial [9]. No primary endpoint was defined for the extension study. Exploratory efficacy endpoints in the extension trial were change from baseline in HbA1c, FPG, body weight, SBP and diastolic blood pressure (DBP) at week 52 and week 76. Other exploratory endpoints were the percentage of patients who were treated in the extension trial and had HbA1c ≥7 % at baseline who reached HbA1c <7 % at week 76 and the use of rescue therapy over 76 weeks. Baseline was defined as the last observed measurement before the first administration of study drug in the initial trial.

Safety was assessed through the reporting of adverse events [AEs; coded using the Medical Dictionary for Drug Regulatory Activities (MedDRA), version 16.0]. AEs of special interest included confirmed hypoglycaemic events (plasma glucose ≤3.9 mmol/l and/or requiring assistance) and AEs consistent with urinary tract infection (UTI), genital infection and volume depletion, which were identified using prospectively defined search categories based on 77, 89 and 8 MedDRA preferred terms, respectively. Changes from baseline in clinical laboratory values at week 76 were also assessed.

### Statistical analyses

No formal sample size calculation was performed for the extension trial; the extension trial was open to all patients who were eligible to participate.

Changes from baseline in HbA1c, FPG, weight, SBP and DBP at week 52 and 76 were analyzed using an analysis of covariance (ANCOVA) model in the full analysis set (FAS: patients who received ≥1 dose of study drug and had a baseline HbA1c measurement in the initial study) with baseline HbA1c and the baseline value of the endpoint in question as linear covariates, and baseline eGFR (MDRD), region and treatment as fixed effects. Data following initiation of rescue therapy were set to missing and missing data were imputed using the last observation carried forward (LOCF) approach. The percentage of patients reaching HbA1c <7 % at week 76 was assessed in patients from the FAS who were treated in the extension trial using a logistic regression model that included treatment, baseline eGFR, region and baseline HbA1c, with non-completers considered failures (patients who did not enter the extension trial were not considered non-completers).

Changes over time in HbA1c, FPG, weight, SBP and DBP were analyzed by means of sensitivity analyses using a restricted maximum likelihood-based mixed model repeated measures (MMRM) approach with baseline HbA1c and the baseline value of the endpoint in question as linear covariates, and baseline eGFR, region, treatment, visit, and visit by treatment interaction as fixed effects, based on observed cases (OC) in the FAS and in FAS-completers. The FAS-completers set comprised patients from the FAS who completed 76 ± 1 weeks’ treatment and had an HbA1c measurement at the week 76 visit. The use of rescue therapy was assessed in the FAS using logistic regression, including treatment as a factor and baseline HbA1c as a covariate.

Safety was assessed in the treated set (patients treated with ≥1 dose of study drug in the initial study) and analyses were descriptive, except for changes from baseline in lipid parameters, which were assessed using ANCOVA with the baseline value and baseline HbA1c as linear covariates, and baseline eGFR and treatment as fixed effects.

## Results

### Patient disposition and characteristics

**Fig. 1:**
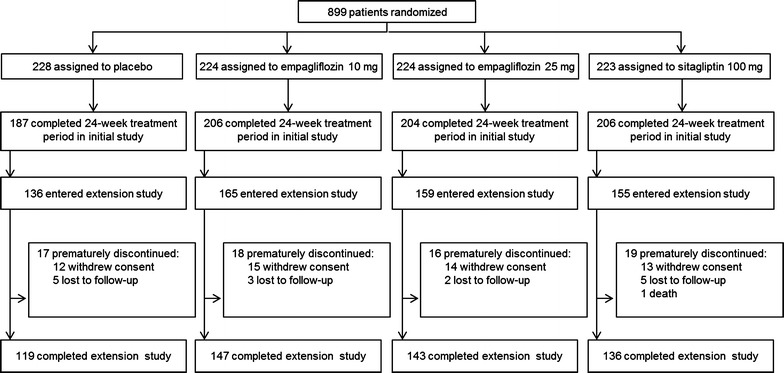
Study flow

The FAS comprised 899 patients. The demographic and baseline characteristics of the FAS were balanced across treatment groups and are summarized in Table 1. Of these 899 patients, 615 (68.4 %) continued in the extension study (Fig. 1). The baseline characteristics of the patients who continued in the extension trial were comparable with the baseline characteristics of the overall patient population treated in the initial 24-week study.

**Table 1:** Baseline characteristics

	Placebo (n = 228)	Empagliflozin 10 mg (n = 224)	Empagliflozin 25 mg (n = 224)	Sitagliptin 100 mg (n = 223)	Total (N = 899)
Male	123 (53.9)	142 (63.4)	145 (64.7)	141 (63.2)	551 (61.3)
Age (years)	54.9 ± 10.9	56.2 ± 11.6	53.8 ± 11.6	55.1 ± 9.9	55.0 ± 11.0
Race					
Asian	146 (64.0)	143 (63.8)	144 (64.3)	143 (64.1)	576 (64.1)
White	76 (33.3)	77 (34.4)	73 (32.6)	76 (34.1)	302 (33.6)
Black/African-American	6 (2.6)	3 (1.3)	7 (3.1)	3 (1.3)	19 (2.1)
Other	0	1 (0.4)	0	1 (0.4)	2 (0.2)
Time since diagnosis of T2DM (years)					
≤1	72 (31.6)	87 (38.8)	91 (40.6)	93 (41.7)	343 (38.2)
>1–5	104 (45.6)	92 (41.1)	83 (37.1)	86 (38.6)	365 (40.6)
>5–10	33 (14.5)	29 (12.9)	37 (16.5)	32 (14.3)	131 (14.6)
>10	19 (8.3)	16 (7.1)	13 (5.8)	12 (5.4)	60 (6.7)
Body weight (kg)	78.2 ± 19.9	78.4 ± 18.7	77.8 ± 18.0	79.3 ± 20.4	78.4 ± 19.2
Body mass index (kg/m^2^)	28.7 ± 6.2	28.3 ± 5.5	28.2 ± 5.5	28.2 ± 5.2	28.4 ± 5.6
HbA1c (%)	7.91 ± 0.78	7.87 ± 0.88	7.86 ± 0.85	7.85 ± 0.79	7.88 ± 0.82
FPG (mmol/l)	8.6 ± 2.0	8.5 ± 1.8	8.5 ± 1.9	8.2 ± 1.6	8.4 ± 1.8
SBP (mmHg)	130.4 ± 16.3	133.0 ± 16.6	129.9 ± 17.5	132.5 ± 15.8	131.4 ± 16.6
DBP (mmHg)	78.9 ± 9.6	79.2 ± 9.6	78.3 ± 9.4	80.1 ± 10.0	79.1 ± 9.6
eGFR (ml/min/1.73 m^2^) (MDRD)	86.8 ± 17.9	87.7 ± 19.2	87.6 ± 18.3	87.6 ± 17.3	87.4 ± 18.2

Data are n (%) or mean ± standard deviation in the full analysis set

### Efficacy

**Fig. 2:**
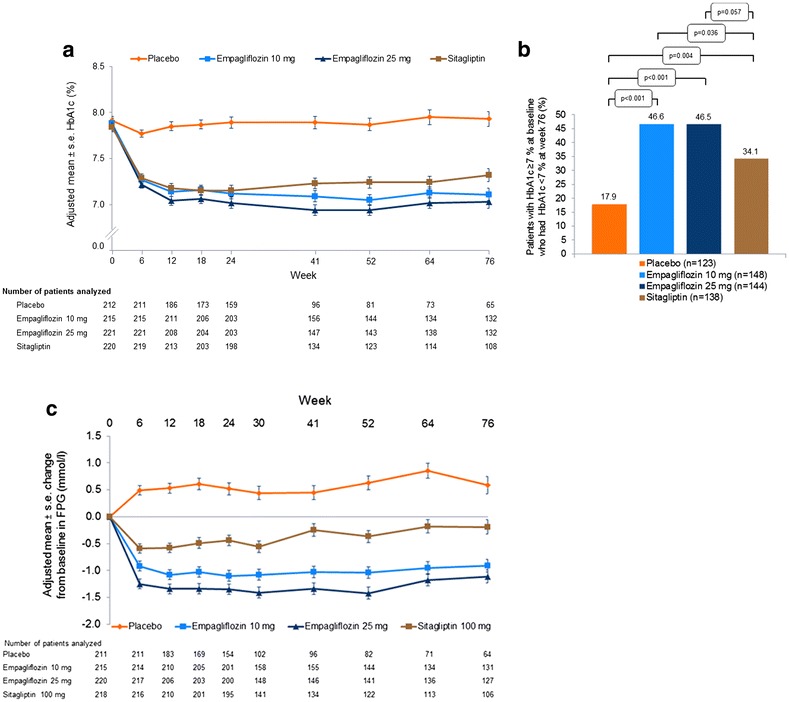
Glycaemic control **a** HbA1c over time [mixed model repeated measures (MMRM) in the full analysis set (FAS), observed cases (OC)]. **b** Patients with HbA1c ≥7 % at baseline who had HbA1c <7 % at week 76 (logistic regression in patients from the FAS treated in the extension trial using non-completers considered failures approach). **c** Change from baseline in FPG over time (MMRM in the FAS, OC). Empagliflozin 10 mg versus placebo odds ratio 4.17 (95 % CI 2.31, 7.51); empagliflozin 25 mg versus placebo odds ratio 3.96 (95 % CI 2.20, 7.14); sitagliptin versus placebo odds ratio 2.42 (95 % CI 1.32, 4.43). Empagliflozin 10 mg versus sitagliptin odds ratio 1.72 (95 % CI 1.04, 2.86); empagliflozin 25 mg versus sitagliptin odds ratio 1.64 (95 % CI 0.99, 2.72)

Reductions in HbA1c at week 76 were greater with both doses of empagliflozin compared with placebo. Differences of adjusted means versus placebo were −0.78 % [95 % confidence interval (CI) −0.94, −0.63] with empagliflozin 10 mg and −0.89 % (−1.04, −0.73) with empagliflozin 25 mg; p < 0.001 for both doses (Table 2). Compared with sitagliptin, adjusted mean changes from baseline in HbA1c at week 76 were greater for empagliflozin 25 mg (differences of adjusted means −0.22 %, 95 % CI −0.38, −0.07, p = 0.005), but not for empagliflozin 10 mg (Table 2). Adjusted mean HbA1c values over 76 weeks are presented in Fig. 2a. The results of sensitivity analyses based on MMRM OC analyses in the FAS and FAS-completers were consistent with those from the ANCOVA LOCF analysis in the FAS (Additional file 1). The proportion of patients treated in the extension trial who had HbA1c ≥7 % at baseline who reached HbA1c <7 % at week 76 was greater with both empagliflozin doses compared with placebo and with empagliflozin 10 mg compared with sitagliptin (Fig. 2b).

**Table 2:** Summary of efficacy results at week 76

	Placebo (n = 228)	Empagliflozin 10 mg (n = 224)	Empagliflozin 25 mg (n = 224)	Sitagliptin 100 mg (n = 223)
HbA1c at week 76 (%)	8.01 ± 0.06	7.22 ± 0.06	7.12 ± 0.06	7.34 ± 0.06
Change from baseline	0.13 ± 0.06	−0.65 ± 0.06	−0.76 ± 0.06	−0.53 ± 0.06
Difference vs. placebo (95 % CI)		−0.78 (−0.94, −0.63)	−0.89 (−1.04, −0.73)	−0.66 (−0.82, −0.51)
p value		<0.001	<0.001	<0.001
Difference vs. sitagliptin (95 % CI)		−0.12 (−0.28, 0.04)	−0.22 (−0.38, −0.07)	
p value		0.131	0.005	
FPG at week 76 (mmol/l)	9.2 ± 0.1	7.5 ± 0.1	7.3 ± 0.1	8.3 ± 0.1
Change from baseline	0.8 ± 0.1	−1.0 ± 0.1	−1.1 ± 0.1	−0.1 ± 0.1
Difference vs. placebo (95 % CI)		−1.8 (−2.1, −1.4)	−1.9 (−2.3, −1.6)	−0.9 (−1.2, −0.6)
p value		<0.001	<0.001	<0.001
Difference vs. sitagliptin (95 % CI)		−0.9 (−1.2, −0.5)	−1.0 (−1.4, −0.7)	
p value		<0.001	<0.001	
Body weight at week 76 (kg)	78.0 ± 0.2	76.2 ± 0.2	76.0 ± 0.2	78.5 ± 0.2
Change from baseline	−0.4 ± 0.2	−2.2 ± 0.2	−2.5 ± 0.2	0.1 ± 0.2
Difference vs. placebo (95 % CI)		−1.8 (−2.4, −1.3)	−2.0 (−2.6, −1.5)	0.5 (0.0, 1.1)
p value		<0.001	<0.001	0.055
Difference vs. sitagliptin (95 % CI)		−2.3 (−2.9, −1.8)	−2.6 (−3.1, −2.0)	
p value		<0.001	<0.001	
SBP at week 76 (mmHg)	130.7 ± 0.8	127.3 ± 0.8	127.3 ± 0.8	131.1 ± 0.8
Change from baseline	−0.7 ± 0.8	−4.1 ± 0.8	−4.2 ± 0.8	−0.3 ± 0.8
Difference vs. placebo (95 % CI)		−3.4 (−5.5, −1.2)	−3.4 (−5.6, −1.2)	0.4 (−1.8, 2.6)
p value		0.003	0.002	0.724
Difference vs. sitagliptin (95 % CI)		−3.7 (−5.9, −1.6)	−3.8 (−6.0, −1.6)	
p value		0.001	0.001	
DBP at week 76 (mmHg)	78.5 ± 0.5	77.5 ± 0.5	77.5 ± 0.5	79.0 ± 0.5
Change from baseline	−0.6 ± 0.5	−1.6 ± 0.5	−1.6 ± 0.5	−0.1 ± 0.5
Difference vs. placebo (95 % CI)		−1.0 (−2.3, 0.4)	−1.0 (−2.4, 0.3)	0.5 (−0.8, 1.9)
p value		0.157	0.132	0.433
Difference vs. sitagliptin (95 % CI)		−1.5 (−2.8, −0.2)	−1.6 (−2.9, −0.2)	
p value		0.029	0.023	

Data are n (%) or adjusted mean ± standard error based on ANCOVA in the FAS (LOCF) unless otherwise indicated

At week 76, reductions in FPG were greater for both empagliflozin doses compared with placebo or sitagliptin (Table 2). Differences of adjusted means versus placebo were −1.8 mmol/l (95 % CI −2.1, −1.4) with empagliflozin 10 mg and −1.9 mmol/l (95 % CI −2.3, −1.6) with empagliflozin 25 mg; p < 0.001 for both doses. Differences of adjusted means versus sitagliptin were −0.9 mmol/l (95 % CI −1.2, −0.5) with empagliflozin 10 mg and −1.0 mmol/l (95 % CI −1.4, −0.7) with empagliflozin 25 mg; p < 0.001 for both doses. Figure 2c shows the adjusted mean changes from baseline in FPG over the 76-week treatment period. The results of sensitivity analyses based on MMRM OC in the FAS and FAS-completers were consistent with findings from the ANCOVA LOCF analysis in the FAS (Additional file 1).

The proportions of patients who received rescue medication up to week 76 were 32.5 % for placebo versus 12.1 % for empagliflozin 10 mg [odds ratio (OR) 0.25, 95 % CI 0.15, 0.41, p < 0.001], 5.8 % for empagliflozin 25 mg (OR 0.11, 95 % CI 0.05, 0.20, p < 0.001) and 15.7 % for sitagliptin (OR 0.37, 95 % CI 0.23, 0.60, p < 0.001). When compared with sitagliptin, ORs were 0.67 (95 % CI 0.38, 1.18, p = 0.163) for empagliflozin 10 mg and 0.29 (95 % CI 0.14, 0.57, p < 0.001) for empagliflozin 25 mg.

**Fig. 3:**
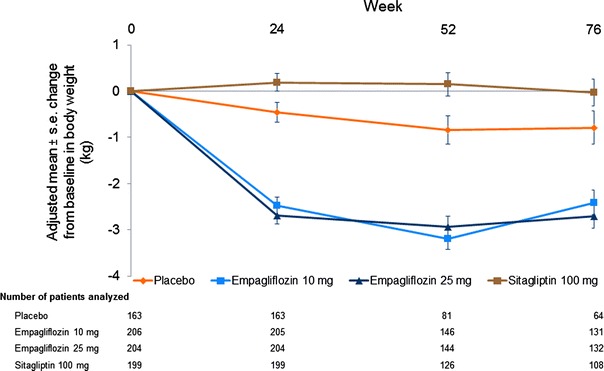
Body weight Change from baseline in body weight over time [mixed model repeated measures (MMRM) in the full analysis set (FAS), observed cases (OC)]

At week 76, empagliflozin 10 mg and 25 mg resulted in a decrease in weight compared with placebo or sitagliptin (Table 2). Differences of adjusted means versus placebo were −1.8 kg (95 % CI −2.4, −1.3) with empagliflozin 10 mg and −2.0 kg (95 % CI −2.6, −1.5) with empagliflozin 25 mg; p < 0.001 for both doses. Differences of adjusted means versus sitagliptin were −2.3 kg (95 % CI −2.9, −1.8) with empagliflozin 10 mg and −2.6 kg (95 % CI −3.1, −2.0) with empagliflozin 25 mg; p < 0.001 for both doses. Adjusted mean changes from baseline in weight over the 76-week trial period are shown in Fig. 3. The results of sensitivity analyses based on MMRM OC (FAS and FAS-completers) were consistent with those from the ANCOVA LOCF analysis in the FAS (Additional Table 1).

Significant reductions in SBP were noted for empagliflozin 10 mg and 25 mg compared with placebo and sitagliptin at week 76 (Table 2). Differences of adjusted means versus placebo were −3.4 mmHg (95 % CI −5.5, −1.2) with empagliflozin 10 mg (p = 0.003) and −3.4 mmHg (95 % CI −5.6, −1.2) with empagliflozin 25 mg (p = 0.002). Differences of adjusted means versus sitagliptin were −3.7 mmHg (95 % CI −5.9, −1.6) with empagliflozin 10 mg and −3.8 mmHg (95 % CI −6.0, −1.6) with empagliflozin 25 mg; p = 0.001 for both doses. Adjusted mean changes from baseline in SBP up to week 76 are presented in Additional file 2. Sensitivity analyses based on MMRM OC in the FAS and FAS-completers showed no difference in adjusted mean change from baseline in SBP between empagliflozin 10 mg or empagliflozin 25 mg and placebo at week 76, but significant differences between both doses of empagliflozin and sitagliptin (Additional file 1).

At week 76, the reductions in DBP with empagliflozin 10 mg and 25 mg were not significantly different compared with placebo, but a significant decrease was noted for both doses when compared with sitagliptin (Table 2). Adjusted mean changes in DBP up to week 76 are presented in Additional file 3. Sensitivity analyses based on MMRM OC in the FAS and FAS-completers showed no difference in adjusted mean change from baseline in DBP for either empagliflozin dose compared with placebo or for empagliflozin 25 mg compared with sitagliptin, but showed a significant difference between empagliflozin 10 mg and sitagliptin at week 76 (Additional file 1).

No clinically meaningful changes in pulse rate were observed. Mean (standard deviation) changes from baseline in pulse rate were −0.2 (9.9), −0.5 (9.0), −1.0 (8.4) and −0.3 (10.1) beats per minute with placebo, empagliflozin 10 mg, empagliflozin 25 mg and sitagliptin, respectively.

The results of exploratory efficacy analyses at week 52 were consistent with those observed at week 76, except that DBP was significantly reduced with empagliflozin 25 mg compared with placebo but not significantly reduced with empagliflozin 10 mg compared with sitagliptin (Additional file 4).

### Safety and tolerability

Median exposure was 17.3, 20.2, 20.2 and 18.1 months in the placebo, empagliflozin 10 mg, empagliflozin 25 mg and sitagliptin groups, respectively. Safety findings are presented in Table 3. The proportion of patients with ≥1 adverse event was similar across the treatment groups. A lower percentage of patients on empagliflozin 10 mg, empagliflozin 25 mg or sitagliptin had AEs leading to discontinuation (4.9, 4.0 and 4.9 %, respectively) compared with placebo (6.6 %). Two patients (0.9 %) per treatment group had confirmed hypoglycaemic AEs; one patient on empagliflozin 10 mg required assistance. Events consistent with UTI were reported in a similar proportion of patients in each treatment group (10.9, 9.4, 9.0, and 9.0 % on placebo, empagliflozin 10 mg, empagliflozin 25 mg, and sitagliptin, respectively) and in a higher proportion of female than male patients. All events consistent with UTI were mild or moderate in intensity except in one patient on empagliflozin 25 mg and one patient on sitagliptin. Events consistent with genital infection were reported in a higher proportion of patients on empagliflozin 10 and 25 mg (5.8 and 6.3 %, respectively) than placebo and sitagliptin (1.7 and 0.9 %, respectively). All events consistent with genital infection were mild or moderate in intensity. Events consistent with volume depletion were reported in 0.4, 2.7, 0.9 and 1.3 % of patients on placebo, empagliflozin 10 mg, empagliflozin 25 mg and sitagliptin, respectively. There was one death in the placebo group and one death in the sitagliptin group (sudden death), which was not regarded by the investigator as related to study drug.

**Table 3:** Adverse events

	Placebo (n = 229)	Empagliflozin 10 mg (n = 224)	Empagliflozin 25 mg (n = 223)	Sitagliptin 100 mg (n = 223)
Any adverse event n (%)	175 (76.4)	172 (76.8)	174 (78.0)	161 (72.2)
Drug-related^a^ adverse events n (%)	36 (15.7)	49 (21.9)	52 (23.3)	31 (13.9)
Discontinuation due to adverse events n (%)	15 (6.6)	11 (4.9)	9 (4.0)	11 (4.9)
Severe adverse events n (%)	14 (6.1)	17 (7.6)	15 (6.7)	17 (7.6)
Serious adverse events n (%)	23 (10.0)	25 (11.2)	16 (7.2)	18 (8.1)
Deaths	1 (0.4)	0 (0.0)	0 (0.0)	1 (0.4)
Adverse events with frequency of ≥5 % in any group (by preferred term) n (%)				
Hyperglycaemia	63 (27.5)	20 (8.9)	11 (4.9)	28 (12.6)
Nasopharyngitis	27 (11.8)	32 (14.3)	25 (11.2)	27 (12.1)
Urinary tract infection	21 (9.2)	20 (8.9)	14 (6.3)	18 (8.1)
Upper respiratory tract infection	12 (5.2)	17 (7.6)	16 (7.2)	19 (8.5)
Dyslipidaemia	15 (6.6)	16 (7.1)	14 (6.3)	14 (6.3)
Back pain	12 (5.2)	7 (3.1)	7 (3.1)	19 (8.5)
Hypertension	13 (5.7)	11 (4.9)	5 (2.2)	14 (6.3)
Bronchitis	10 (4.4)	11 (4.9)	6 (2.7)	12 (5.4)
Diarrhoea	9 (3.9)	12 (5.4)	6 (2.7)	8 (3.6)
Special interest categories n (%)				
Confirmed hypoglycaemia^b^	2 (0.9)	2 (0.9)	2 (0.9)	2 (0.9)
Events requiring assistance	0 (0.0)	1 (0.4)	0 (0.0)	0 (0.0)
Events consistent with urinary tract infection^c^	25 (10.9)	21 (9.4)	20 (9.0)	20 (9.0)
Male	4 (3.2)	4 (2.8)	4 (2.8)	6 (4.3)
Female	21 (20.0)	17 (20.7)	16 (20.3)	14 (17.1)
Events consistent with genital infection^d^	4 (1.7)	13 (5.8)	14 (6.3)	2 (0.9)
Male	2 (1.6)	4 (2.8)	4 (2.8)	1 (0.7)
Female	2 (1.9)	9 (11.0)	10 (12.7)	1 (1.2)
Events consistent with volume depletion^e^	1 (0.4)	6 (2.7)	2 (0.9)	3 (1.3)

Data from the treated set

^a^As reported by the investigator

^b^Plasma glucose ≤3.9 mmol/l and/or requiring assistance

^c^Based on 77 preferred terms

^d^Based on 89 preferred terms

^e^Based on eight preferred terms

Small increases in haematocrit and eGFR and small decreases in serum uric acid were observed in the empagliflozin groups (Additional file 5). Compared with placebo, there was an increase from baseline in low density lipoprotein (LDL) cholesterol and total cholesterol in patients treated with empagliflozin 25 mg and in high density lipoprotein (HDL) cholesterol in both empagliflozin dose groups. No differences versus placebo in change from baseline in triglycerides or LDL/HDL cholesterol ratio were noted for either empagliflozin dose (Table 4).

**Table 4:** Lipid parameters

	Placebo		Empagliflozin 10 mg		Empagliflozin 25 mg		Sitagliptin 100 mg	
	Baseline	Change from baseline at week 76	Baseline	Change from baseline at week 76	Baseline	Change from baseline at week 76	Baseline	Change from baseline at week 76
Total cholesterol (mmol/l)	5.03 ± 0.08	−0.03 ± 0.05	5.00 ± 0.08	0.10 ± 0.05	5.00 ± 0.08	0.24 ± 0.05	4.95 ± 0.07	0.05 ± 0.05
Difference vs. placebo				0.12 ± 0.07		0.27 ± 0.07		0.08 ± 0.07
p value				0.091		<0.001		0.272
Difference vs. sitagliptin				0.04 ± 0.07		0.19 ± 0.07		
p value				0.558		0.012		
HDL cholesterol (mmol/l)	1.26 ± 0.02	0.03 ± 0.01	1.24 ± 0.02	0.11 ± 0.01	1.25 ± 0.02	0.12 ± 0.01	1.26 ± 0.02	0.02 ± 0.01
Difference vs. placebo				0.08 ± 0.02		0.09 ± 0.02		−0.01 ± 0.02
p value				<0.001		<0.001		0.647
Difference vs. sitagliptin				0.09 ± 0.02		0.09 ± 0.02		
p value				<0.001		<0.001		
LDL cholesterol (mmol/l)	2.90 ± 0.06	−0.04 ± 0.04	2.86 ± 0.07	0.03 ± 0.05	2.75 ± 0.07	0.15 ± 0.04	2.74 ± 0.05	0.07 ± 0.04
Difference vs. placebo				0.07 ± 0.06		0.19 ± 0.06		0.11 ± 0.06
p value				0.248		0.002		0.088
Difference vs. sitagliptin				−0.04 ± 0.06		0.09 ± 0.06		
p value				0.581		0.174		
LDL/HDL cholesterol ratio	2.43 ± 0.07	−0.11 ± 0.04	2.40 ± 0.06	−0.12 ± 0.04	2.32 ± 0.06	−0.05 ± 0.04	2.30 ± 0.05	0.00 ± 0.04
Difference vs. placebo				−0.01 ± 0.06		0.06 ± 0.06		0.12 ± 0.06
p value				0.921		0.256		0.040
Difference vs. sitagliptin				−0.12 ± 0.06		−0.05 ± 0.06		
p value				0.033		0.358		
Triglycerides (mmol/l)	2.01 ± 0.09	−0.03 ± 0.11	2.08 ± 0.12	−0.13 ± 0.11	2.37 ± 0.20	−0.02 ± 0.11	2.20 ± 0.13	−0.07 ± 0.11
Difference vs. placebo				−0.10 ± 0.16		0.01 ± 0.16		−0.04 ± 0.16
p value				0.532		0.931		0.795
Difference vs. sitagliptin				−0.06 ± 0.16		0.06 ± 0.16		
p value				0.717		0.730		

Baseline data are mean ± standard error, change from baseline data are adjusted mean ± standard error, based on ANCOVA in the full analysis set (last observation carried forward)

*HDL* high density lipoprotein, *LDL* low density lipoprotein

## Discussion

This extension study showed that treatment with empagliflozin 10 mg or 25 mg or sitagliptin for 76 weeks led to sustained improvements in glycaemic control compared with placebo. When compared with sitagliptin, empagliflozin 25 mg reduced HbA1c, and both empagliflozin doses reduced FPG. Furthermore, empagliflozin 10 mg and 25 mg led to sustained weight loss compared with placebo or sitagliptin. The weight loss observed after 76 weeks’ treatment with empagliflozin is clinically meaningful as weight management remains a major challenge in the treatment of patients with T2DM [18] and weight gain with glucose-lowering medication is a concern for the majority of patients [19, 20]. A study of empagliflozin 25 mg given as add-on to metformin for 104 weeks found that nearly 90 % of the weight loss with empagliflozin was due to a reduction in fat mass, and that empagliflozin reduced both abdominal visceral adipose tissue and abdominal subcutaneous adipose tissue [13]. The initial weight loss seen with SGLT2 inhibitors may be due to the mild osmotic effects associated with UGE, but sustained weight loss is believed to result from loss of calories through UGE.

In previous Phase III studies, empagliflozin reduced SBP compared with placebo [8–12, 15, 21]. In this study, empagliflozin showed a reduction in SBP compared with placebo and sitagliptin at week 76 based on ANCOVA analyses with LOCF imputation, but no reductions with empagliflozin compared with placebo across the sensitivity analyses (based on MMRM, OC). This discrepant result may be explained by the higher rate of early discontinuation and the greater need for rescue therapy in the placebo group.

The sustained effects of empagliflozin on glycaemic control and weight in this extension trial were consistent with the results of a study in which patients received empagliflozin monotherapy for 90 weeks [22]. The effects of sitagliptin on glycaemic control and weight in this study were comparable with those observed in previous studies of sitagliptin monotherapy in drug-naïve patients with T2DM [23–25].

Recently published results of the EMPA-REG OUTCOME^®^ trial have shown that in patients with type 2 diabetes and high cardiovascular risk (i.e., established cardiovascular disease), empagliflozin added to standard of care reduced the primary composite outcome of cardiovascular death, non-fatal myocardial infarction or non-fatal stroke (3-point major adverse cardiovascular events); cardiovascular death; hospitalisation for heart failure; and overall mortality compared to placebo [21, 26]. Although the mechanisms behind the observed effects of empagliflozin in this patient population are not yet understood, they may involve reductions in hyperglycaemia, blood pressure and weight as well as effects on plasma volume and sodium retention [27]; and reductions in arterial stiffness [28, 29].

Both doses of empagliflozin and sitagliptin were well tolerated. In accordance with its insulin-independent mode of action [30], and the results of previous studies, empagliflozin monotherapy was not associated with an increased risk of hypoglycaemia. In line with previous trials of SGLT2 inhibitors, events consistent with genital infection were reported in a greater proportion of patients treated with empagliflozin than with placebo, and were more common in female than male patients [31]. Consistent with previous studies of empagliflozin [9–12, 21], there was no higher risk of UTI in patients treated with empagliflozin in this study.

Strengths of the design of this study include the long duration, that treatment remained double-blind throughout the extension period and the inclusion of a sitagliptin group as an active comparator. Limitations of this study include that all endpoints, while pre-specified, were exploratory, with no primary endpoint defined for the extension study, and the amount of missing data. Only 68 % of patients randomized in the initial 24-week study entered the extension period; however, this is within the range for extension studies of other SGLT2 inhibitors [32–34]. Data obtained after initiation of rescue therapy (5.8–32.5 % across treatment groups) were set to missing and imputed. Overall, 51.4 % of HbA1c data at week 76 were imputed using a LOCF approach. The methods for handling missing data were analyzed by means of sensitivity analyses, which revealed consistent results regarding improvements in glycaemic control and weight loss with empagliflozin. A further limitation of this study was that the results may not be generalizable to all patients with T2DM, as for example, approximately two-thirds of the patients in the trial were Asian.

In conclusion, results from this extension study indicate that empagliflozin 10 mg and 25 mg given as monotherapy to drug-naïve patients with T2DM leads to sustained improvements in glycaemic control and reductions in weight compared with placebo and sustained reductions in HbA1c (for empagliflozin 25 mg), weight and SBP compared with sitagliptin over 76 weeks.

## Additional files

10.1186/s12933-015-0314-0 Sensitivity analyses of efficacy endpoints at week 76.
10.1186/s12933-015-0314-0 Change from baseline in systolic blood pressure over time.
10.1186/s12933-015-0314-0 Change from baseline in diastolic blood pressure over time.
10.1186/s12933-015-0314-0 Summary of efficacy results at week 52.
10.1186/s12933-015-0314-0 Clinical laboratory parameters except lipids.

## Abbreviations

AE: adverse events
ANCOVA: analysis of covariance
CI: confidence interval
DBP: diastolic blood pressure
DPP-4: dipeptidyl peptidase-4
FAS: full analysis set
FPG: fasting plasma glucose
eGFR: estimated glomerular filtration rate
HbA1c: haemoglobin A1c
HDL: high density lipoprotein
LDL: low density lipoprotein
LOCF: last observation carried forward
MDRD: modification of diet in renal disease formula
MedDRA: Medical Dictionary for Drug Regulatory Activities
MMRM: mixed model repeated measures
OC: observed cases
OR: odds ratio
SGLT2: sodium glucose cotransporter 2
SE: standard error
SBP: systolic blood pressure
T2DM: type 2 diabetes mellitus
UGE: urinary glucose excretion
UTI: urinary tract infection
