# Supplementary material for: Safety, tolerability and effects on cardiometabolic risk factors of empagliflozin monotherapy in drug-naïve patients with type 2 diabetes: a double-blind extension of a Phase III randomized controlled trial
Source: Cardiovasc Diabetol. 2015 Dec 23;14:154. doi: 10.1186/s12933-015-0314-0 (PMC4690334; doi:10.1186/s12933-015-0314-0)
Supplement: Supplementary file 1 — 10.1186/s12933-015-0314-0 Sensitivity analyses of efficacy endpoints at week 76. [file 12933_2015_314_MOESM1_ESM.docx]

**Additional files to accompany manuscript “Empagliflozin monotherapy in drug-naïve patients with type 2 diabetes: a double-blind extension of a Phase III randomized controlled trial” by M Roden et al**

**Additional file 1 Sensitivity analyses of efficacy endpoints at week 76**

|  | **Placebo**  **(n=228)** | **Empagliflozin 10 mg**  **(n=224)** | **Empagliflozin  25 mg**  **(n=224)** | **Sitagliptin**  **100 mg**  **(n=223)** |
| --- | --- | --- | --- | --- |
| **MMRM in FAS (OC)** | | | | |
| HbA1c, n* | 65 | 132 | 132 | 108 |
| Change from baseline in HbA1c at week 76, % | 0.13 ± 0.08 | -0.70 ± 0.07 | -0.77 ± 0.07 | -0.48 ± 0.07 |
| Difference vs. placebo (95% CI) |  | -0.82 (-1.04, -0.61) | -0.90 (-1.11, -0.69) | -0.61 (-0.83, -0.40) |
| p-value |  | <0.001 | <0.001 | <0.001 |
| Difference vs. sitagliptin (95% CI) |  | -0.21 (-0.41, -0.02) | -0.29 (-0.48, -0.09) |  |
| p-value |  | 0.032 | 0.004 |  |
| FPG, n* | 64 | 131 | 127 | 106 |
| Change from baseline in FPG at week 76, mmol/l | 0.6 ± 0.2 | -0.9 ± 0.1 | -1.1 ± 0.1 | -0.2 ± 0.1 |
| Difference vs. placebo (95% CI) |  | -1.5 (-1.9, -1.1) | -1.7 (-2.1, -1.3) | -0.8 (-1.2, -0.4) |
| p-value |  | <0.001 | <0.001 | <0.001 |
| Difference vs. sitagliptin (95% CI) |  | -0.7 (-1.1, -0.4) | -0.9 (-1.3, -0.6) |  |
| p-value |  | <0.001 | <0.001 |  |
| Body weight, n* | 64 | 131 | 132 | 108 |
| Change from baseline in body weight at week 76, kg | -0.8 ± 0.4 | -2.4 ± 0.3 | -2.7 ± 0.3 | -0.0 ± 0.3 |
| Difference vs. placebo (95% CI) |  | -1.6 (-2.5, -0.7) | -1.9 (-2.8, -1.0) | 0.8 (-0.1, 1.7) |
| p-value |  | <0.001 | <0.001 | 0.100 |
| Difference vs. sitagliptin (95% CI) |  | -2.4 (-3.2, -1.6) | -2.7 (-3.4, -1.9) |  |
| p-value |  | <0.001 | <0.001 |  |
| SBP, n* | 64 | 131 | 131 | 108 |
| Change from baseline in SBP at week 76, mmHg | -2.8 ± 1.3 | -4.8 ± 0.9 | -4.4 ± 0.9 | 0.5 ± 1.0 |
| Difference vs. placebo (95% CI) |  | -2.0 (-5.1, 1.1) | -1.7 (-4.8, 1.5) | 3.2 (0.0, 6.4) |
| p-value |  | 0.206 | 0.299 | 0.051 |
| Difference vs. sitagliptin (95% CI) |  | -5.2 (-8.0, -2.5) | -4.9 (-7.6, -2.1) |  |
| p-value |  | <0.001 | <0.001 |  |
| DBP, n* | 64 | 131 | 131 | 108 |
| Change from baseline in DBP at week 76, mmHg | -2.2 ± 0.8 | -2.0 ± 0.6 | -1.5 ± 0.6 | 0.2 ± 0.7 |
| Difference vs. placebo (95% CI) |  | 0.2 (-1.8, 2.2) | 0.7 (-1.4, 2.7) | 2.4 (0.3, 4.5) |
| p-value |  | 0.860 | 0.518 | 0.026 |
| Difference vs. sitagliptin (95% CI) |  | -2.2 (-4.0, -0.4) | -1.7 (-3.5, 0.1) |  |
| p-value |  | 0.015 | 0.058 |  |
| **MMRM in FAS-completers (OC)** | | | | |
| HbA1c, n* | 64 | 131 | 131 | 107 |
| Change from baseline in HbA1c at week 76, % | -0.05 ± 0.09 | -0.73 ± 0.07 | -0.84 ± 0.07 | -0.58 ± 0.07 |
| Difference vs. placebo (95% CI) |  | -0.67 (-0.89, -0.45) | -0.78 (-1.01, -0.56) | -0.53 (-0.76, -0.31) |
| p-value |  | <0.001 | <0.001 | <0.001 |
| Difference vs. sitagliptin (95% CI) |  | -0.14 (-0.34, 0.06) | -0.25 (-0.45, -0.05) |  |
| p-value |  | 0.167 | 0.015 |  |
| FPG, n* | 63 | 130 | 126 | 105 |
| Change from baseline in FPG at week 76, mmol/l | 0.3 (0.2) | -0.9 (0.1) | -1.2 (0.1) | -0.4 (0.1) |
| Difference vs. placebo (95% CI) |  | -1.3 (-1.7, -0.9) | -1.6 (-2.0, -1.2) | -0.7 (-1.2, -0.3) |
| p-value |  | <0.001 | <0.001 | <0.001 |
| Difference vs. sitagliptin (95% CI) |  | -0.5 (-0.9, -0.2) | -0.8 (-1.2, -0.5) |  |
| p-value |  | 0.004 | <0.001 |  |
| Body weight, n* | 63 | 130 | 130 | 107 |
| Change from baseline in body weight at week 76, kg | -0.7 ± 0.4 | -2.5 ± 0.3 | -2.8 ± 0.3 | -0.0 ± 0.3 |
| Difference vs. placebo (95% CI) |  | -1.8 (-2.7, -0.9) | -2.1 (-3.0, -1.1) | 0.7 (-0.3, 1.7) |
| p-value |  | <0.001 | <0.001 | 0.147 |
| Difference vs. sitagliptin (95% CI) |  | -2.5 (-3.3, -1.7) | -2.8 (-3.6, -2.0) |  |
| p-value |  | <0.001 | <0.001 |  |
| SBP, n* | 63 | 130 | 130 | 107 |
| Change from baseline in SBP at week 76, mmHg | -3.3 ± 1.3 | -5.2 ± 1.0 | -4.9 ± 1.0 | 0.5 ± 1.1 |
| Difference vs. placebo (95% CI) |  | -1.9 (-5.2, 1.4) | -1.6 (-4.9, 1.7) | 3.8 (0.4, 7.2) |
| p-value |  | 0.266 | 0.332 | 0.029 |
| Difference vs. sitagliptin (95% CI) |  | -5.7 (-8.6, -2.8) | -5.4 (-8.3, -2.5) |  |
| p-value |  | <0.001 | <0.001 |  |
| DBP, n* | 63 | 130 | 130 | 107 |
| Change from baseline in DBP at week 76, mmHg | -2.4 ± 0.9 | -2.5 ± 0.6 | -1.5 ± 0.6 | 0.0 ± 0.7 |
| Difference vs. placebo (95% CI) |  | 0.0 (-2.2, 2.1) | 0.9 (-1.2, 3.0) | 2.4 (0.2, 4.6) |
| p-value |  | 0.985 | 0.399 | 0.030 |
| Difference vs. sitagliptin (95% CI) |  | -2.5 (-4.3, -0.6) | -1.5 (-3.4, 0.3) |  |
| p-value |  | 0.010 | 0.108 |  |

Data are adjusted mean ± standard error unless otherwise indicated and are based on restricted maximum likelihood-based mixed model repeated measures (MMRM) in full analysis set (FAS) and FAS-completers (observed cases [OC]).
* Number of analyzed patients.

HbA1c: haemoglobin A1c; CI: confidence interval; FPG: fasting plasma glucose; SBP: systolic blood pressure; DBP: diastolic blood pressure.
