# Supplementary material for: Safety, tolerability and effects on cardiometabolic risk factors of empagliflozin monotherapy in drug-naïve patients with type 2 diabetes: a double-blind extension of a Phase III randomized controlled trial
Source: Cardiovasc Diabetol. 2015 Dec 23;14:154. doi: 10.1186/s12933-015-0314-0 (PMC4690334; doi:10.1186/s12933-015-0314-0)
Supplement: Supplementary file 3 — 10.1186/s12933-015-0314-0 Change from baseline in diastolic blood pressure over time. [file 12933_2015_314_MOESM3_ESM.docx]

**Additional files to accompany manuscript “Empagliflozin monotherapy in drug-naïve patients with type 2 diabetes: a double-blind extension of a Phase III randomized controlled trial” by M Roden et al**

**Additional file 3** **Change from baseline in diastolic blood pressure over time.**

A


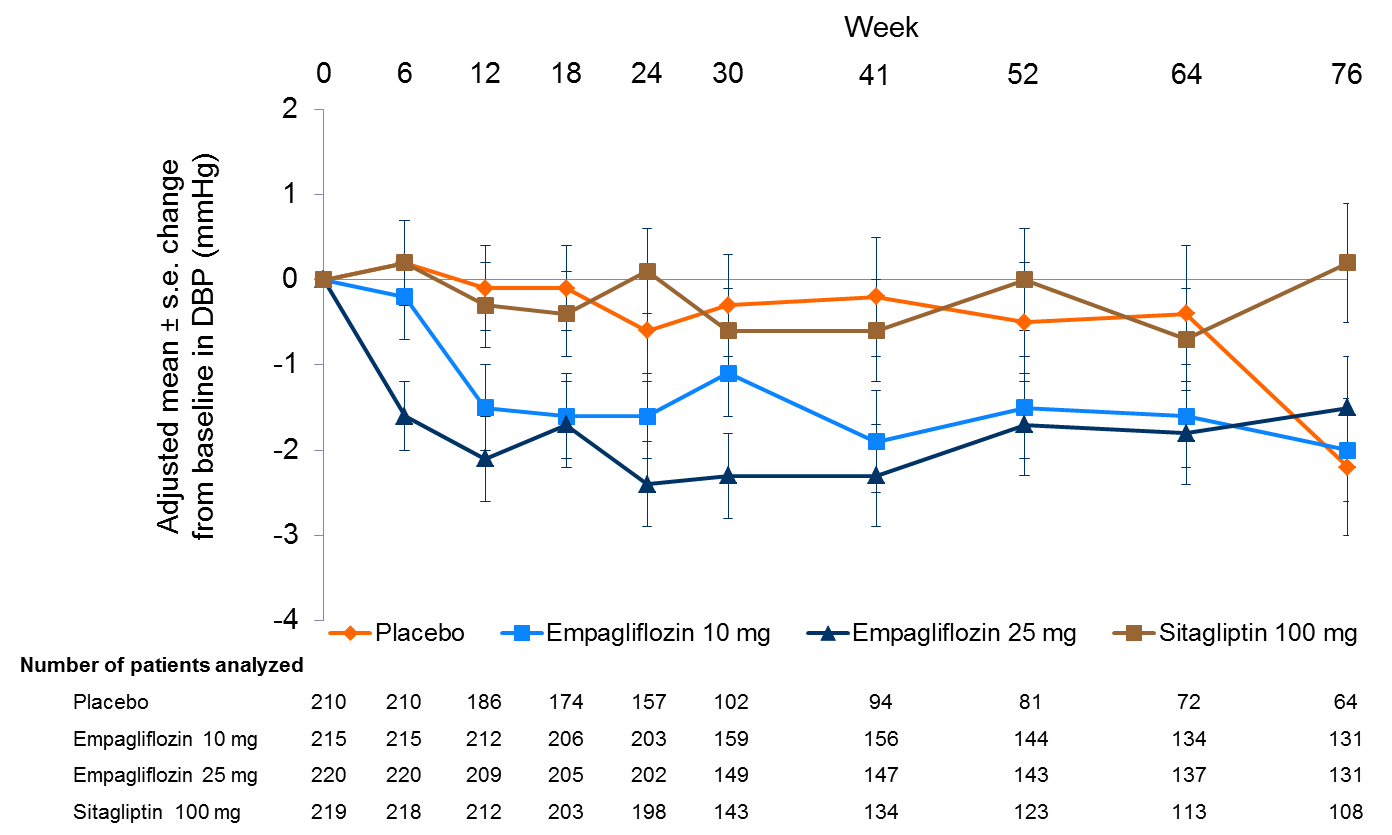


Mixed model repeated measures in the full analysis set, observed cases.

s.e.: standard error; DBP: diastolic blood pressure.
