# Supplementary material for: Safety, tolerability and effects on cardiometabolic risk factors of empagliflozin monotherapy in drug-naïve patients with type 2 diabetes: a double-blind extension of a Phase III randomized controlled trial
Source: Cardiovasc Diabetol. 2015 Dec 23;14:154. doi: 10.1186/s12933-015-0314-0 (PMC4690334; doi:10.1186/s12933-015-0314-0)
Supplement: Supplementary file 4 — 10.1186/s12933-015-0314-0 Summary of efficacy results at week 52. [file 12933_2015_314_MOESM4_ESM.docx]

**Additional files to accompany manuscript “Empagliflozin monotherapy in drug-naïve patients with type 2 diabetes: a double-blind extension of a Phase III randomized controlled trial” by M Roden et al**

**Additional file 4 Summary of efficacy results at week 52**

|  | **Placebo**  **(n=228)** | **Empagliflozin 10 mg**  **(n=224)** | **Empagliflozin  25 mg**  **(n=224)** | **Sitagliptin**  **100 mg**  **(n=223)** |
| --- | --- | --- | --- | --- |
| HbA1c at week 52, % | 7.97 ± 0.05 | 7.18 ± 0.05 | 7.06 ± 0.05 | 7.30 ± 0.05 |
| Change from baseline | 0.09 ± 0.05 | −0.70 ± 0.05 | −0.82 ± 0.05 | −0.58 ± 0.05 |
| Difference vs. placebo (95% CI) |  | -0.79 (-0.94, -0.64) | -0.91 (-1.06, -0.76) | -0.67 (-0.82, -0.52) |
| p-value |  | <0.001 | <0.001 | <0.001 |
| Difference vs. sitagliptin (95% CI) |  | -0.12 (-0.27, 0.03) | -0.24 (-0.39, -0.09) |  |
| p-value |  | 0.125 | 0.002 |  |
| FPG at week 52, mmol/l | 9.2 ± 0.1 | 7.4 ± 0.1 | 7.1 ± 0.1 | 8.2 ± 0.1 |
| Change from baseline | 0.7 ± 0.1 | -1.1 ± 0.1 | -1.3 ± 0.1 | -0.2 ± 0.1 |
| Difference vs. placebo (95% CI) |  | -1.8 (-2.1, -1.5) | -2.1 (-2.4, -1.8) | -1.0 (-1.3, -0.6) |
| p-value |  | <0.001 | <0.001 | <0.001 |
| Difference vs. sitagliptin (95% CI) |  | -0.8 (-1.1, -0.5) | -1.1 (-1.4, -0.8) |  |
| p-value |  | <0.001 | <0.001 |  |
| Body weight at week 52, kg | 77.9 ± 0.2 | 75.7 ± 0.2 | 75.8 ± 0.2 | 78.6 ± 0.2 |
| Change from baseline | -0.5 ± 0.2 | -2.7 ± 0.2 | -2.6 ± 0.2 | 0.1 ± 0.2 |
| Difference vs. placebo (95% CI) |  | -2.2 (-2.7, -1.7) | -2.1 (-2.7, -1.6) | 0.6 (0.1, 1.1) |
| p-value |  | <0.001 | <0.001 | 0.022 |
| Difference vs. sitagliptin (95% CI) |  | -2.8 (-3.4, -2.3) | -2.8 (-3.3, -2.2) |  |
| p-value |  | <0.001 | <0.001 |  |
| SBP at week 52, mmHg | 131.2 ± 0.8 | 126.6 ± 0.8 | 127.0 ± 0.8 | 129.8 ± 0.8 |
| Change from baseline | -0.2 ± 0.8 | -4.9 ± 0.8 | -4.5 ± 0.8 | -1.6 ± 0.8 |
| Difference vs. placebo (95% CI) |  | -4.6 (-6.8, -2.5) | -4.2 (-6.4, -2.1) | -1.4 (-3.5, 0.8) |
| p-value |  | <0.001 | <0.001 | 0.211 |
| Difference vs. sitagliptin (95% CI) |  | -3.3 (-5.4, -1.1) | -2.8 (-5.0, -0.7) |  |
| p-value |  | 0.003 | 0.011 |  |
| DBP at week 52, mmHg | 79.0 ± 0.5 | 77.9 ± 0.5 | 77.2 ± 0.5 | 78.8 ± 0.5 |
| Change from baseline | -0.2 ± 0.5 | -1.3 ± 0.5 | -1.9 ± 0.5 | -0.3 ± 0.5 |
| Difference vs. placebo (95% CI) |  | -1.1 (-2.4, 0.2) | -1.7 (-3.1, -0.4) | -0.2 (-1.5, 1.2) |
| p-value |  | 0.106 | 0.011 | 0.826 |
| Difference vs. sitagliptin (95% CI) |  | -0.9 (-2.3, 0.4) | -1.6 (-2.9, -0.2) |  |
| p-value |  | 0.166 | 0.021 |  |

Data are n (%) or adjusted mean ± standard error based on ANCOVA in the full analysis set (last observation carried forward) unless otherwise indicated.

HbA1c: haemoglobin A1c; CI: confidence interval; FPG: fasting plasma glucose; SBP: systolic blood pressure; DBP: diastolic blood pressure.
