# Supplementary material for: Safety, tolerability and effects on cardiometabolic risk factors of empagliflozin monotherapy in drug-naïve patients with type 2 diabetes: a double-blind extension of a Phase III randomized controlled trial
Source: Cardiovasc Diabetol. 2015 Dec 23;14:154. doi: 10.1186/s12933-015-0314-0 (PMC4690334; doi:10.1186/s12933-015-0314-0)
Supplement: Supplementary file 5 — 10.1186/s12933-015-0314-0 Clinical laboratory parameters except lipids. [file 12933_2015_314_MOESM5_ESM.docx]

**Additional files to accompany manuscript “Empagliflozin monotherapy in drug-naïve patients with type 2 diabetes: a double-blind extension of a Phase III randomized controlled trial” by M Roden et al**

**Additional file 5 Clinical laboratory parameters other than lipids**

|  | **Placebo** | | **Empagliflozin 10 mg** | | **Empagliflozin 25 mg** | | **Sitagliptin 100 mg** | |
| --- | --- | --- | --- | --- | --- | --- | --- | --- |
|  | **Baseline** | **Change from baseline*** | **Baseline** | **Change from baseline*** | **Baseline** | **Change from baseline*** | **Baseline** | **Change from baseline*** |
| Haematocrit, %^†^ | 43.4 ± 4.7 | -0.3 ± 3.2 | 43.6 ± 4.4 | 3.1 ± 4.0 | 43.8 ± 4.7 | 3.1 ± 3.9 | 43.5 ± 4.3 | 0.0 ± 3.6 |
| Uric acid, μmol/l^†^ | 307 ± 133 | -4 ± 85 | 293 ± 109 | -53 ± 85 | 297 ± 124 | -59 ± 89 | 298 ± 114 | 14 ± 80 |
| Creatinine, μmol/l^†^ | 79 ± 12 | -1 ± 6 | 79 ± 12 | -1 ± 12 | 80 ± 11 | -2 ± 8 | 79 ± 11 | -0 ± 8 |
| eGFR, ml/min/1.73 m^2^ (MDRD) | 86.8 ± 17.9 | 0.6 ± 9.7 | 87.7 ± 19.2 | 2.6 ± 13.7 | 87.5 ± 18.3 | 2.1 ± 11.6 | 87.6 ± 17.3 | 0.0 ± 12.0 |
| Electrolytes, mmol/l^†^ |  |  |  |  |  |  |  |  |
| Sodium | 141 ± 2 | 0 ± 2 | 141 ± 2 | 1 ± 2 | 141 ± 2 | 0 ± 2 | 141 ± 2 | 0 ± 2 |
| Potassium | 4.1 ± 0.3 | 0.1 ± 0.3 | 4.1 ± 0.3 | -0.0 ± 0.3 | 4.0 ± 0.3 | -0.0 ± 0.3 | 4.1 ± 0.3 | 0.0 ± 0.3 |
| Calcium | 2.4 ± 0.1 | 0 ± 0.1 | 2.4 ± 0.1 | -0.0 ± 0.1 | 2.4 ± 0.1 | -0.0 ± 0.1 | 2.4 ± 0.1 | -0.0 ± 0.1 |
| Magnesium | 1.0 ± 0.1 | -0.0 ± 0.1 | 1.0 ± 0.1 | 0.0 ± 0.1 | 1.0 ± 0.1 | 0.0 ± 0.1 | 1.0 ± 0.1 | -0.0 ± 0.1 |
| Phosphate | 1.2 ± 0.1 | 0.0 ± 0.1 | 1.2 ± 0.1 | 0.0 ± 0.1 | 1.2 ± 0.1 | 0.0 ± 0.1 | 1.2 ± 0.1 | -0.0 ± 0.1 |

Data are mean ± standard deviation in the treated set. *Change from baseline at last value on treatment for haematocrit, creatinine, uric acid and electrolytes; change from baseline at week 76 for eGFR. ^†^Normalized to a standard reference range.

eGFR: estimated glomerular filtration rate; MDRD: Modification of Diet in Renal Disease.
